# Association of Survival With Chemoendocrine Therapy in Women With Small, Hormone Receptor–Positive, ERBB2-Positive, Node-Negative Breast Cancer

**DOI:** 10.1001/jamanetworkopen.2020.2507

**Published:** 2020-04-09

**Authors:** Sung Jun Ma, Oluwadamilola T. Oladeru, Anurag K. Singh

**Affiliations:** 1 Department of Radiation Medicine, Roswell Park Comprehensive Cancer Center, Buffalo, New York; 2 Department of Radiation Oncology, Massachusetts General Hospital, Boston

## Abstract

This cohort study examines the association of survival with adjuvant chemoendocrine therapy for hormone receptor–positive, ERBB2-positive, node-negative breast cancer.

## Introduction

Small node-negative, hormone receptor (HR)–positive, ERBB2 (previously HER2/neu)–positive tumors represent a heterogenous category of breast cancer, with recurrence rates ranging from less than 5% up to 25% at 5 years, with or without adjuvant treatments.^1,2^ The current National Comprehensive Cancer Network guideline^3^ acknowledges the lack of representation of T1a and T1b tumors in prior randomized trials and, thus, recommends the consideration of chemotherapy for tumors 1 cm or smaller at the discretion of clinicians. In the absence of randomized trial data on the role of chemotherapy for small tumors, the cutoff size at which chemotherapy should be omitted remains unclear. Using a national-level hospital registry, we conducted an observational cohort study to address this knowledge gap.

## Methods

The US National Cancer Database was queried for female patients with HR-positive, ERBB2-positive, pT1a-bN0 breast cancer diagnosed between 2010 and 2015 who received hormone therapy with or without chemotherapy. Ethical approval was waived by the Roswell Park Comprehensive Cancer Center institutional review board, because the National Cancer Database is a deidentified data set. This study follows the Strengthening the Reporting of Observational Studies in Epidemiology (STROBE) reporting guideline.

The Kaplan-Meier method and Cox multivariable analysis were performed to analyze overall survival. Propensity score matching was based on the nearest neighbor method in a 1:1 ratio without a replacement. The standardized difference between variables was less than 0.1, indicating appropriate matching.^4^ All *P* values were 2-sided, with *P* < .05 considered statistically significant. R statistical software version 3.6.1 (R Project for Statistical Computing) was used for all analyses. Data analysis was performed from November 2019 to January 2020. Additional details are shown in the eAppendix in the Supplement.

## Results

A total of 10 065 patients (median [interquartile range] age, 59 [51-67] years) were identified, including 5346 patients who received chemotherapy and 4719 patients who did not (ERBB2-directed therapy was coded distinctly from chemotherapy during 2013 to 2015, and only 15% of such patients underwent either chemotherapy or ERBB2-directed therapy alone; data not shown) (Table). The median (interquartile range) follow-up was 41.8 (24.3-62.6) months. On multivariable analysis, multiagent chemotherapy was associated with improved overall survival (hazards ratio [HR], 0.69; 95% CI, 0.52-0.90; *P* = .006), and tumor size as a continuous variable was associated with worse mortality (for every 1-mm increase, HR, 1.07; 95% CI, 1.03-1.12; *P* = .002). There was a statistically significant interaction between multiagent chemotherapy and tumor size (*P *for interaction = .02). Cox multivariable analysis was repeated with each tumor size cutoff ranging from 2 mm to 9 mm, and an 8-mm cutoff was statistically significant (*P* for interaction = .01), with a large effect size and narrow 95% CI on subgroup analysis. Multiagent chemotherapy was not associated with improved overall survival for tumors smaller than 8 mm (HR, 1.00; 95% CI, 0.70-1.43; *P* = .99), compared with tumors 8 mm to 10 mm, which favors the use of chemotherapy (HR, 0.53; 95% CI, 0.36-0.78; *P* = .001). Similar findings were observed in 1641 and 648 matched pairs, respectively (tumors <8 mm, HR, 0.88; 95% CI, 0.58-1.34; *P* = .55; tumors 8-10 mm, HR, 0.48; 95% CI, 0.27-0.85; *P* = .01) (Figure).

**Table.** Baseline Characteristics of Matched Cohorts

Characteristic	Participants, No. (%) (N = 2289)					
	Tumor size <8 mm (n = 1641)			Tumor size 8-10 mm (n = 648)		
	Chemotherapy		*P* value	Chemotherapy		*P* value
	No	Yes		No	Yes	
Facility volume						
Low	127 (8)	126 (8)	.99	48 (7)	57 (9)	.28
Intermediate	367 (22)	364 (22)		168 (26)	146 (23)	
High	1147 (70)	1151 (70)		432 (67)	445 (69)	
Total	1641 (100)	1641 (100)		648 (100)	648 (100)	
Facility type						
Nonacademic	1035 (63)	1021 (62)	.08	462 (71)	459 (71)	.68
Academic	534 (33)	519 (32)		178 (27)	184 (28)	
Not available	72 (4)	101 (6)		8 (1)	5 (1)	
Total	1641 (100)	1641 (100)		648 (100)	648 (100)	
Age, y						
<50	477 (29)	488 (30)	.88	86 (13)	84 (13)	.97
50-74	1139 (69)	1126 (69)		529 (82)	532 (82)	
≥75	25 (2)	27 (2)		33 (5)	32 (5)	
Total	1641 (100)	1641 (100)		648 (100)	648 (100)	
Charlson-Deyo Comorbidity Index score						
0	1420 (87)	1432 (87)	.63	566 (87)	566 (87)	.68
1	170 (10)	167 (10)		68 (10)	72 (11)	
2	51 (3)	42 (3)		14 (2)	10 (2)	
Total	1641 (100)	1641 (100)		648 (100)	648 (100)	
Annual income						
Above median	1124 (68)	1147 (70)	.71	441 (68)	433 (67)	.68
Below median	512 (31)	490 (30)		207 (32)	214 (33)	
Not available	5 (0)	4 (0)		0 (0)	1 (0)	
Total	1641 (100)	1641 (100)		648 (100)	648 (100)	
Tumor histologic profile						
Ductal or lobular	1641 (100)	1641 (100)	NA	647 (100)	648 (100)	>.99
Others	0 (0)	0 (0)		1 (0)	0 (0)	
Total	1641 (100)	1641 (100)		648 (100)	648 (100)	
Tumor grade						
Differentiated			.62			.50
Well	144 (9)	120 (7)		92 (14)	81 (13)	
Moderately	823 (50)	847 (52)		372 (57)	398 (61)	
Poorly	566 (34)	567 (35)		150 (23)	135 (21)	
Others	5 (0)	4 (0)		0 (0)	0 (0)	
Not available	103 (6)	103 (6)		34 (5)	34 (5)	
Total	1641 (100)	1641 (100)		648 (100)	648 (100)	
Race						
White	1358 (83)	1370 (83)	.82	569 (88)	571 (88)	.97
Black	163 (10)	165 (10)		54 (8)	55 (8)	
Others	105 (6)	93 (6)		21 (3)	19 (3)	
Not available	15 (1)	13 (1)		4 (1)	3 (0)	
Total	1641 (100)	1641 (100)		648 (100)	648 (100)	
Year						
2010-2012	874 (53)	868 (53)	.86	306 (47)	322 (50)	.40
2013-2015	767 (47)	773 (47)		342 (53)	326 (50)	
Total	1641 (100)	1641 (100)		648 (100)	648 (100)	
Lymph nodes examined, No.						
0-2	844 (51)	834 (51)	.77	368 (57)	385 (59)	.23
>2	788 (48)	795 (48)		279 (43)	259 (40)	
Not available	9 (1)	12 (1)		1 (0)	4 (1)	
Total	1641 (100)	1641 (100)		648 (100)	648 (100)	
Hormone receptor status						
Estrogen receptor positive and progesterone receptor positive	1158 (71)	1161 (71)	.94	501 (77)	499 (77)	.95
Estrogen receptor positive or progesterone receptor positive	483 (29)	480 (29)		147 (23)	149 (23)	
Total	1641 (100)	1641 (100)		648 (100)	648 (100)	
Surgery						
Lumpectomy	956 (58)	965 (59)	.78	445 (69)	446 (69)	>.99
Mastectomy	685 (42)	676 (41)		202 (31)	202 (31)	
Others	0 (0)	0 (0)		1 (0)	0 (0)	
Total	1641 (100)	1641 (100)		648 (100)	648 (100)	
Margin						
Negative	1582 (96)	1574 (96)	.76	632 (98)	638 (98)	.34
Positive	55 (3)	62 (4)		12 (2)	9 (1)	
Not available	4 (0)	5 (0)		4 (1)	1 (0)	
Total	1641 (100)	1641 (100)		648 (100)	648 (100)	
Radiation therapy						
None	667 (41)	655 (40)	.67	216 (33)	215 (33)	.80
External beam	907 (55)	926 (56)		395 (61)	402 (62)	
Others	67 (4)	59 (4)		36 (6)	31 (5)	
Not available	0 (0)	1 (0)		1 (0)	0 (0)	
Total	1641 (100)	1641 (100)		648 (100)	648 (100)	
Radiation dose, median, (interquartile range), Gy	60 (52.4-60.8)	6.4 (53.5-61.0)	.13	60 (52.6-60.8)	60 (54.0-60.8)	.62
Readmission within 30 d						
None	1564 (95)	1541 (94)	.24	623 (96)	619 (96)	.74
Unplanned	13 (1)	21 (1)		3 (0)	5 (1)	
Planned	38 (2)	52 (3)		11 (2)	15 (2)	
Others	0 (0)	1 (0)		1 (0)	0 (0)	
Not available	26 (2)	26 (2)		10 (2)	9 (1)	
Total	1641 (100)	1641 (100)		648 (100)	648 (100)	
Postoperative inpatient stay duration, median (interquartile range), d	0 (0-2.0)	0 (0-2.0)	.38	0 (0-1.0)	0 (0-1.0)	.50

Abbreviation: NA, not applicable.

**Figure.**
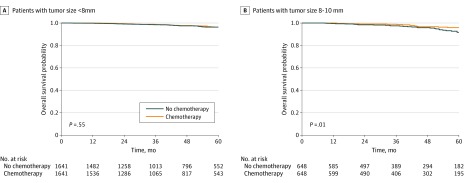
Kaplan-Meier Survival Curves After Matching Matching was performed for variables, including treatment facility volume, facility type, age, race, income level, comorbidity score, year of diagnosis, histologic profile, tumor grade, number of lymph nodes examined, hormone receptor status, type of surgery and radiation, surgical margin, radiation dose, postoperative readmissions, and duration of postoperative inpatient admission. Graphs show survival curves for patients with tumors smaller than 8 mm (A) and tumors 8 to 10 mm (B) who did or did not receive chemotherapy.

## Discussion

To our knowledge, this is the first report to suggest that there is an association between improved survival and adjuvant chemoendocrine therapy specifically for HR-positive, ERBB2-positive tumors 8 mm to 10 mm compared with those smaller than 8 mm. It is evident that tumors 10 mm and smaller represent a heterogeneous group whose treatment should be tailored to improve the risk-to-benefit ratio of systemic therapy. We acknowledge the inherent challenges of diagnostic concordance in the context of millimeter-based decisions, which underscores the importance of expert pathology review. Our study is limited by the lack of specific systemic therapy regimens. Therapy directed at ERBB2 was coded distinctly from chemotherapy during 2013 to 2015, and only 15% of such patients underwent either chemotherapy or ERBB2-directed therapy alone (data not shown). Subgroup analysis using this cohort would be difficult because of the small sample sizes, as neither systemic therapy alone is a definitive recommendation by National Comprehensive Cancer Network in this setting.^3^ Postoperative readmissions and duration of postoperative inpatient admission as proxy measures for postoperative performance status were well balanced after matching.^5^ Nevertheless, while we await results of prospective trials, including the ATEMPT trial (ClinicalTrials.gov identifier, NCT01853748), our data can help clinicians in decision-making on adjuvant systemic therapy for patients with small HR-positive, ERBB2-positive breast cancers.

## Notes

Supplement. **eAppendix.** Supplemental Methods
