# Supplementary material for: Association of Survival With Chemoendocrine Therapy in Women With Small, Hormone Receptor–Positive, ERBB2-Positive, Node-Negative Breast Cancer
Source: JAMA Netw Open. 2020 Apr 9;3(4):e202507. doi: 10.1001/jamanetworkopen.2020.2507 (PMC7146098; doi:10.1001/jamanetworkopen.2020.2507)
Supplement: Supplement. — eAppendix. Supplemental Methods [file jamanetwopen-3-e202507-s001.pdf]

Supplementary Online Content

Ma SJ, Oladeru OT, Singh AK. Association of survival with chemoendocrine therapy in women with small, hormone receptor–positive, ERBB2-positive, node-negative breast cancer. *JAMA Netw Open*. 2020;3(4):e202507.
doi:10.1001/jamanetworkopen.2020.2507

eAppendix. Supplemental Methods

This supplementary material has been provided by the authors to give readers additional information about their work.

© 2020 Ma SJ et al. *JAMA Network Open.*

eAppendix. Supplemental Methods

All missing values were coded as unknown. Pertinent prognostic factors, such as performance status, type of chemotherapy, toxicity, and tumor progression or recurrence, were not captured in the NCDB. Primary endpoint was overall survival (OS), defined as the time interval between diagnosis and the last follow-up or death.

Kaplan-Meier method and log-rank tests were used to evaluate OS. Comparison of categorical and continuous variables were performed using Fisher exact test and Mann-Whitney U test, respectively. When performing Cox proportional hazard multivariable analysis (MVA), the MVA model was initially constructed based on all statistically significant variables from the Cox univariable analysis, and a backward stepwise elimination was used to finalize the model.

Potential interaction between the use of chemotherapy and tumor size was assessed by adding interaction terms to Cox proportional hazard MVA final model. If the interaction term was statistically significant, the final model was re-evaluated for each tumor size cutoff ranging from 2 to 9 mm. The tumor size cutoff of interest was selected based on its largest effect size with narrow width of confidence interval and corresponding p value.

In addition, to address the selection bias, propensity score matching was performed based on baseline patient and tumor variables, including age, race, comorbidity score, histology, grade, year of diagnosis, facility volume, facility type, surgery, surgical margin, radiation, and postoperative readmission and inpatient stay.

© 2020 Ma SJ et al. *JAMA Network Open.*
